# Diagnostic value of Kaiser score combined with breast vascular assessment from breast MRI for the characterization of breast lesions

**DOI:** 10.3389/fonc.2023.1165405

**Published:** 2023-07-06

**Authors:** Xin-zhu Zhou, Lian-hua Liu, Shuang He, Hui-fang Yao, Li-ping Chen, Chen Deng, Shuang-Ling Li, Xiao-yong Zhang, Hua Lai

**Affiliations:** ^1^ Department of Radiology, Chengdu Women's and Children's Central Hospital, School of Medicine, University of Electronic Science and Technology of China, Chengdu, China; ^2^ Clinical Science, Philips Healthcare, Chengdu, China

**Keywords:** breast, breast neoplasms, magnetic resonance imaging, clinical decision making, breast cancer

## Abstract

**Objectives:**

The Kaiser scoring system for breast magnetic resonance imaging is a clinical decision-making tool for diagnosing breast lesions. However, the Kaiser score (KS) did not include the evaluation of breast vascularity. Therefore, this study aimed to use KS combined with breast vascular assessment, defined as KS^*^, and investigate the effectiveness of KS^*^ in differentiating benign from malignant breast lesions.

**Methods:**

This retrospective study included 223 patients with suspicious breast lesions and pathologically verified results. The histopathological diagnostic criteria were according to the fifth edition of the WHO classification of breast tumors. The KS^*^ was obtained after a joint evaluation combining the original KS and breast vasculature assessment. The receiver operating characteristic (ROC) curve was used for comparing differences in the diagnostic performance between KS^*^ and KS, and the area under the receiver operating characteristic (AUC) was compared.

**Results:**

There were 119 (53.4%) benign and 104 (46.6%) malignant lesions in total. The overall sensitivity, specificity, and accuracy of increased ipsilateral breast vascularity were 69.2%, 76.5%, and 73.1%, respectively. The overall sensitivity, specificity, and accuracy of AVS were 82.7%, 76.5%, and 79.4%, respectively. For all lesions included the AUC of KS^*^ was greater than that of KS (0.877 vs. 0.858, P = 0.016). The largest difference in AUC was observed in the non-mass subgroup (0.793 vs. 0.725, P = 0.029).

**Conclusion:**

Ipsilaterally increased breast vascularity and a positive AVS sign were significantly associated with malignancy. KS combined with breast vascular assessment can effectively improve the diagnostic ability of KS for breast lesions, especially for non-mass lesions.

## Highlights

1. Ipsilaterally increased vascularity and AVS were significantly associated with malignancy.
2. The Kaiser score had high efficiency in the differential diagnosis of breast lesions, especially mass lesions.
3. The combination of breast vascular assessment and Kaiser score could be better than Kaiser score in diagnosing breast cancer, especially for non-mass lesions.

## Introduction

Breast magnetic resonance imaging is an important diagnostic tool for detecting pathological abnormalities in the breast, especially for lesions that are ambiguous or inconclusive on mammography or ultrasonography. Compared with mammography and ultrasonography, breast MRI has a high sensitivity for detecting lesions (1). However, the diagnostic efficacy of breast MRI varies greatly and is related to breast enhancement patterns and kinetics (2). The American College of Radiology (ACR) Breast Imaging Reporting and Data System (BI-RADS) is widely used in the interpretation of breast images and provides uniform terminology and a standardized classification for breast lesions. However, translating specific image characteristics into diagnostic categories based on ACR-BI-RADS vocabulary is not always easy. Accurate diagnosis of breast diseases using ACR-BI-RADS may require a lot of working experience, so it is a challenge for young radiologists. Additionally, those lesions that do not exhibit explicit malignant characteristics but display suspicious manifestations are classified as BI-RADS 4 (>2% but <95% likelihood of malignancy) (3). Such a wide range of probabilities urges patients to undergo unnecessary tissue biopsies (4). It is necessary to seek a new systematization method beyond BI-RADS to improve the diagnostic classification of breast MRI in the assessment of breast lesions. It may be feasible to establish systematic methods using artificial intelligence or deep learning (5–7).

The Kaiser scoring system for breast MRI is an evidence-based clinical decision-making tool for the categorization of breast lesions (distinguishing benign from malignant lesions). The Kaiser score (KS), combining independent diagnostic BI-RADS lexicon criteria in a comprehensible flowchart, consists of 11 rating categories ranging from 1 to 11, with each category corresponding to a distinct likelihood of malignancy, which simplifies the classification and categorization of lesions (8). Previous studies also demonstrated the diagnostic value and reliability of KS in assessing breast lesions, which could achieve a better diagnostic efficiency than ACR-BI-RADS (9–11). Compared with ACR-BI-RADS, KS can effectively differentiate malignant lesions from benign lesions, avoiding more unnecessary biopsies (10, 12, 13). Furthermore, KS could improve inter-reader agreement, improving the diagnostic efficacy of less experienced radiologists (14). However, the diagnostic efficacy of KS is insufficient in diagnosing non-mass breast lesions compared with mass lesions, potentially due to the morphological complexity of non-mass lesions, suggesting that KS still needs further improvement (12, 15).

The importance of tumor neovascularization has been emphasized in breast cancer clinical trials (16). Therefore, in addition to the morphological characteristics of breast lesions, the evaluation of the blood supply to the lesions is also of great significance for their qualitative diagnosis. Increased ipsilateral breast vascularization and the presence of the adjacent vessel sign (AVS) were associated with breast cancer, which indicate a poor prognosis (17–20). A previous study showed that after adjusting the ACR-BI-RADS category with the breast vascularity score, the diagnostic accuracy of breast MRI was significantly increased (21).

Therefore, this study aimed to propose using a composite score (modified KS, defined as KS^*^) by integrating the Kaiser score with breast vascular assessment to differentiate benign from malignant breast lesions and compare the diagnostic efficacy of KS* and KS for breast lesions.

## Materials and methods

### Study patients

**Figure 1:**
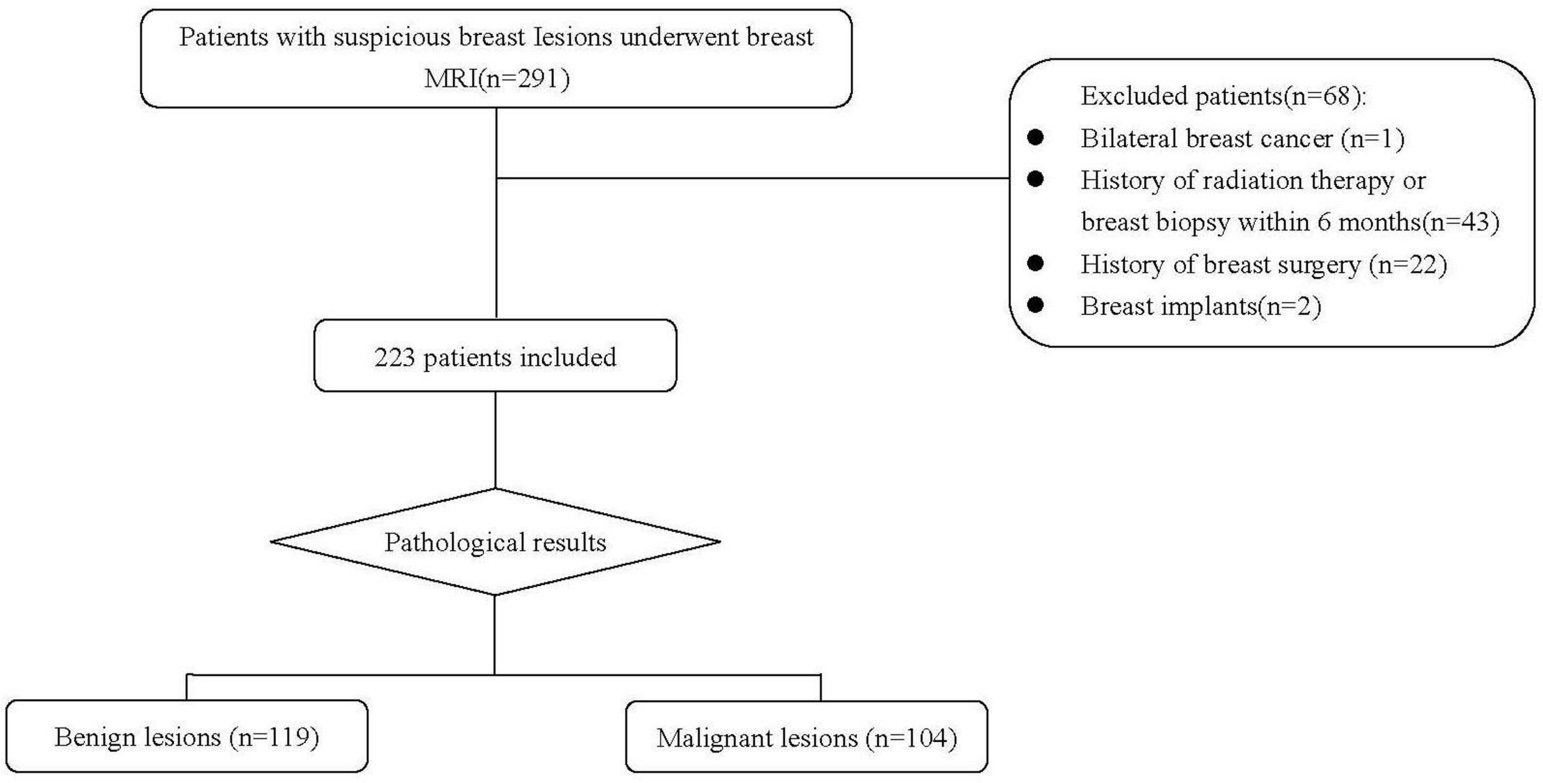
Patient selection flowchart.

The retrospective study was approved by our institutional review board, and informed consent was waived. A total of 291 consecutive patients who underwent breast MRI from September 2018 to May 2021 in our hospital due to suspicious lesions detected by mammography, ultrasound, or clinical examination were included in the study. Patients with unilateral breast lesions and pathologically verified results were considered for analysis. All patients underwent a breast MRI within 15 days of surgery or biopsy. The exclusion criteria for this study were as follows: (1) patients with bilateral breast cancer (n = 1); (2) patients with a history of radiation therapy or breast biopsy within 6 months (n = 43); (3) patients with a history of breast surgery (n = 22); and (4) patients who had breast implants (n = 2). Finally, 223 patients were included for the analysis. Patient selection criteria are detailed in **Figure 1**.

### MRI protocol

All patients underwent breast MRI with a 3T MRI scanner (Ingenia, Philips, Amsterdam, the Netherlands) using a dedicated 7-channel phased-array breast coil. The axial protocol started with a pre-contrast T1-weighted turbo spin echo (T1w-TSE), a T2-weighted spectral pre-saturation attenuated inversion recovery (T2w-SPAIR), and a diffusion-weighted imaging (DWI) sequence. Axial T1-weighted dynamic sequences were measured once before and five times after the contrast agent was injected with 83 s/phase temporal resolution. Gadolinium-diethylene triamine pentaacetate (Gd-DTPA) was administered intravenously at a dose of 0.1 mmol/kg body weight and a flow rate of 2 ml/s, followed by flushing with 20 ml of saline. Details of the MRI protocol are given in **Table 1**. Unenhanced images from the dynamic sequence were subtracted from the second series of contrast-enhanced images to generate the subtracted images, and maximum intensity projection (MIP) reconstruction was applied to the subtracted images.

**Table 1:** Breast magnetic resonance imaging protocols.

Sequence	TR/TE (ms)	FOV (mm^2^)	Matrix	Slice thickness (mm)	Spatial resolution (mm^3^)	Scanning time
T1w-TSE	400/8	280 × 340	280 × 340	5	1 × 1 × 5	1 min, 3 s
T2w-SPAIR	5,000/65	280 × 340	280 × 340	5	1 × 1 × 5	3 min, 30 s
DWI	12,500/90	340 × 340	170 × 170	5	2 × 2 × 5	3 min, 8 s
T1 dynamic	4.4/2.1	250 × 350	250 × 350	0.75	1 × 1 × 0.75	8 min, 18 s

### Image interpretation

All imaging datasets were independently analyzed by two radiologists blinded to histological results (reader 1 with 4 years of breast MRI diagnostic experience and reader 2 with 6 years of breast MRI diagnostic experience). Discrepancies between the two observers’ interpretations were resolved by consensus.

For breast vasculature assessment, the vessel that had a length of ≥3 cm and a diameter of ≥2 mm was counted for the breast with the lesion and its contralateral breast on MIP images. If the number of vessels in the lesion-bearing breast was greater by two or more than those in the contralateral breast, it was considered increased ipsilateral breast vascularity (17). Adjacent vessel sign (AVS) was defined as the presence of vessels either entering the lesion or in contact with the edge of the lesion, which was clearly delineated on any of the subtraction images (19).

The determination of KS was based on five diagnostic features (lesion type, shape of margins, root sign, enhancement kinetics, and presence of edema). The KS of lesions ranges from 1 to 11 (an increased Kaiser score reflects increasing probabilities of malignancy) (8). The KS^*^ was obtained by combining the breast vasculature evaluation, and the diagnostic assessment was made based on the KS^*^. If a lesion with a score less than or equal to 7 showed increased ipsilateral breast vascularity and the presence of AVS simultaneously, the KS was increased by 3. The range of modified KS* was 1–11.

### Histopathological analysis

All lesions identified as BI-RADS 4 (suspicious) were either surgically biopsied or underwent image-guided biopsies in accordance with pre-established standards (22–24). All histopathological diagnoses were performed by board-certified breast pathologists. The diagnostic criteria for histopathology were according to the fifth edition of the WHO classification of breast tumors (25). Malignant lesions were defined as the presence of invasive cancer or ductal carcinoma *in situ* found after a needle biopsy or surgery. Surgical biopsies were carried out on all patients who had malignant lesions, lesions with uncertain malignant potential, and lesions with inconsistent radiological and pathological results. If benign lesions were found histopathologically, the patient underwent breast MRI follow-up.

### Statistical analysis

Statistical analysis was performed using SPSS software (version 23.0) and MedCalc software (version 20.03). Categorical variables were expressed as a percentage (%) and compared using the chi-square test. Continuous variables were expressed as the mean ± standard deviation. The Kappa test was used to test consistency intra and inter two observers (kappa values below 0.4, between 0.4 and 0.7, and above 0.7 were considered bad, good, and excellent) (26). Sensitivity, specificity, and accuracy of increased ipsilateral breast vascularity and positive AVS signs for mass and non-mass lesions were compared using the chi-square test. To assess the diagnostic accuracy in distinguishing benign from malignant lesions, the area under the receiver operating characteristics curve (AUC) of KS and KS^*^ was calculated and compared using the DeLong test. Two-tailed *p*-values of <0.05 were considered statistically significant.

## Results

### Patients and lesions

The mean age of the patients included in the analysis was 45.1 ± 11.0 years (range, 11–73 years). There were 119 (53.4%) benign and 104 (46.6%) malignant lesions. The most common benign lesion was fibroadenoma (42, 35.2%), and the most common malignant lesion was invasive ductal carcinoma (86, 82.7%). A total of 164 (73.5%) mass lesions and 59 (26.5%) non-mass lesions were found, among which 85 (51.8%) of the mass lesions were malignant and 19 (32.2%) of the non-mass lesions were malignant. The size of non-mass lesions (mean, 36.7 mm; range, 10–90 mm; median, 32 mm) was larger than that of mass lesions (mean, 25.8 mm; range, 6–128 mm; median, 21 mm; P = 0.001). Detailed pathological diagnoses and subtypes of benign and malignant lesions are given in **Table 2**.

**Table 2:** Final histological characteristics of the included lesions.

	Subtypes	Total (%)	Mass lesions (%)	Non-mass lesions (%)
Benign		119/223 (53.4%)	79/164 (48.2%)	40/59 (67.8%)
	Adenosis, sclerosing adenosis	36 (30.2%)	20 (25.3%)	16 (40.0%)
	Fibroadenoma	42 (35.2%)	41 (51.9%)	1 (2.5%)
	Inflammation	14 (11.7%)	5 (6.3%)	9 (22.5%)
	Papilloma	24 (20.1%)	11 (13.9%)	13 (32.5%)
	Benign phylloid tumor	2 (2%)	2 (2.5%)	0
	Radial scar	1 (0.8%)	0	1 (2.5%)
Malignant		104/223 (46.6%)	85/164 (51.8%)	19/59 (32.2%)
	DCIS	13 (12.5%)	3 (3.5%)	10 (52.6%)
	Invasive ductal carcinoma	86 (82.7%)	78 (91.8%)	8 (42.1%)
	Invasive solid papillary carcinoma	3 (2.9%)	2 (2.3%)	1 (5.3%)
	Invasive micropapillary carcinoma	2 (1.9%)	2 (2.3%)	0

DCIS, ductal carcinoma *in situ*.

### Diagnostic accuracy of increased ipsilateral breast vascularity and presence of AVS

As shown in **Table 3**, the measurements of increased ipsilateral breast vascularity and AVS all had excellent consistency intra and inter two observers (all kappa values >0.7). One hundred of 223 (44.8%) patients had increased ipsilateral breast vascularity. The positive AVS sign was observed in 114 of 223 (51.1%) lesions. For all lesions, the sensitivity, specificity, and accuracy of increased ipsilateral breast vascularity were 69.2%, 76.5%, and 73.1%, respectively. The sensitivity, specificity, and accuracy of AVS were 82.7%, 76.5%, and 79.4%, respectively. A total of 80 lesions showed increased ipsilateral breast vascularity and a positive AVS sign at the same time, of which 64 (80%) were malignant and 16 (20%) were benign, and the sensitivity, specificity, and accuracy of the combination of the above two variables were 61.5%, 85.7%, and 74.4%, respectively.

**Table 3:** Analysis of intra observer and inter observer consistency.

	Intra observer consistency		Inter observer consistency	
	Kappa value	95% CI	Kappa value	95% CI
Increased ipsilateral breast vascularity	0.955	0.916–0.994	0.900	0.842–0.958
AVS	0.973	0.958–0.988	0.964	0.946–0.982

The increased ipsilateral breast vascularity and positive AVS sign in the mass lesions were comparable to those in the non-mass lesions (increased ipsilateral breast vascularity: 44.8% (74/165) vs. 45.6% (26/57), P = 0.66; AVS sign: 53.3% (88/165) vs. 45.6% (26/57), P = 0.36). The specificity and accuracy of the positive AVS sign for the mass lesions were greater than those for the non-mass lesions (82.2% (65/79) vs. 65.0% (26/40), P = 0.036; 83.5% (137/164) vs. 67.8% (40/59), P = 0.010, respectively). The diagnostic value of increased ipsilateral breast vascularity and a positive AVS sign for the mass and non-mass lesions is shown in **Table 4**.

**Table 4:** Diagnostic efficiency of increased ipsilateral breast vascularity and positive AVS as signs of malignancy in different types of lesions.

	All lesions	Mass lesions (n = 164)	Non-mass lesions (n = 59)	P
Increased ipsilateral breast vascularity				
Sensitivity	69.2% (72/104)	65.9% (56/85)	84.2% (16/19)	0.118
Specificity	76.5% (91/119)	78.5% (62/79)	72.5% (29/40)	0.467
Accuracy	73.1% (163/223)	71.9% (118/164)	76.2% (45/59)	0.521
PPV	72.0% (72/100)	76.7% (56/73)	59.3% (16/27)	0.084
NPV	74.0% (91/123)	68.1% (62/91)	90.6% (29/31)	0.005
AVS				
Sensitivity	82.7% (86/104)	84.7% (72/85)	73.7% (14/19)	0.251
Specificity	76.5% (91/119)	82.2% (65/79)	65.0% (26/40)	0.036
Accuracy	79.4% (177/223)	83.5% (137/164)	67.8% (40/59)	0.010
PPV	75.4% (86/114)	83.7% (72/86)	50.0% (14/28)	<0.001
NPV	83.5% (91/109)	83.3% (65/78)	83.7% (26/31)	0.946

AVS, adjacent vessel sign.

### Influence of the adjusted KS based on breast vascular assessment on lesion diagnosis

**Figure 2:**
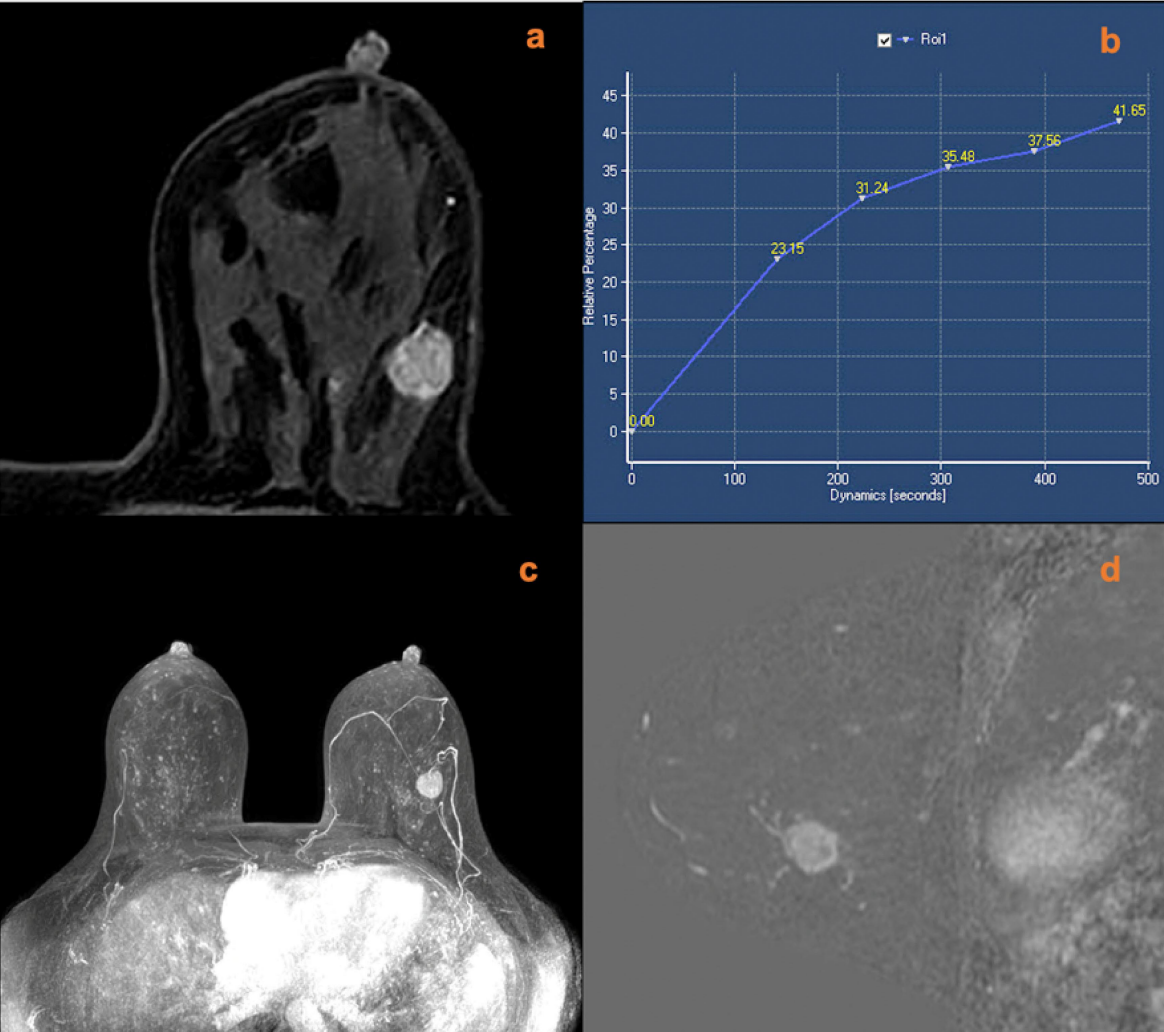
A 51-year-old female was newly identified with a left breast mass lesion for 10 days. MRI (**A**, contrast-enhanced axial; **B**, time signal intensity curve; **C**, maximum intensity projections image; **D**, subtraction image) shows a left breast mass lesion with an irregular margin and persistent enhancement. The lesion also increased the ipsilateral breast vascularity and the positive AVS sign. The KS of the lesions was 3, while KS^*^ was 6. Postoperative pathology confirmed that the lesion was an invasive ductal carcinoma.

**Figure 3:**
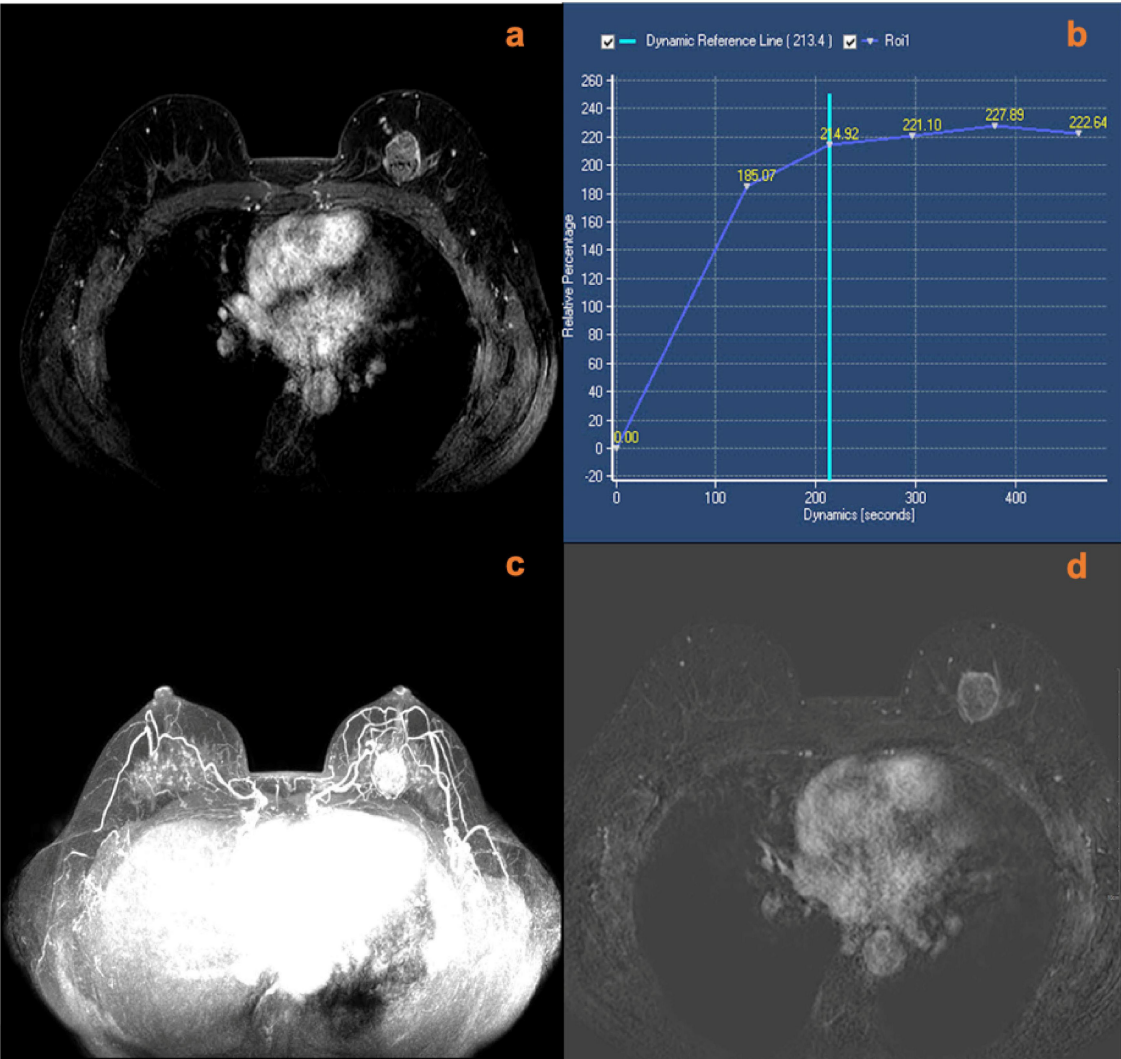
** **A 49-year-old female was identified with a left breast mass lesion for 3 months. MRI (**A**, contrast-enhanced axial; **B**, time signal intensity curve; **C**, maximum intensity projections image; **D**, subtraction image) shows a left breast mass lesion with an irregular margin and plateau enhancement. The lesion also increased ipsilateral breast vascularity and had a positive AVS sign. The KS of the lesions was 5, while KS^*^ was 8. Postoperative pathology confirmed that the lesion was an invasive ductal carcinoma.

**Figure 4:**
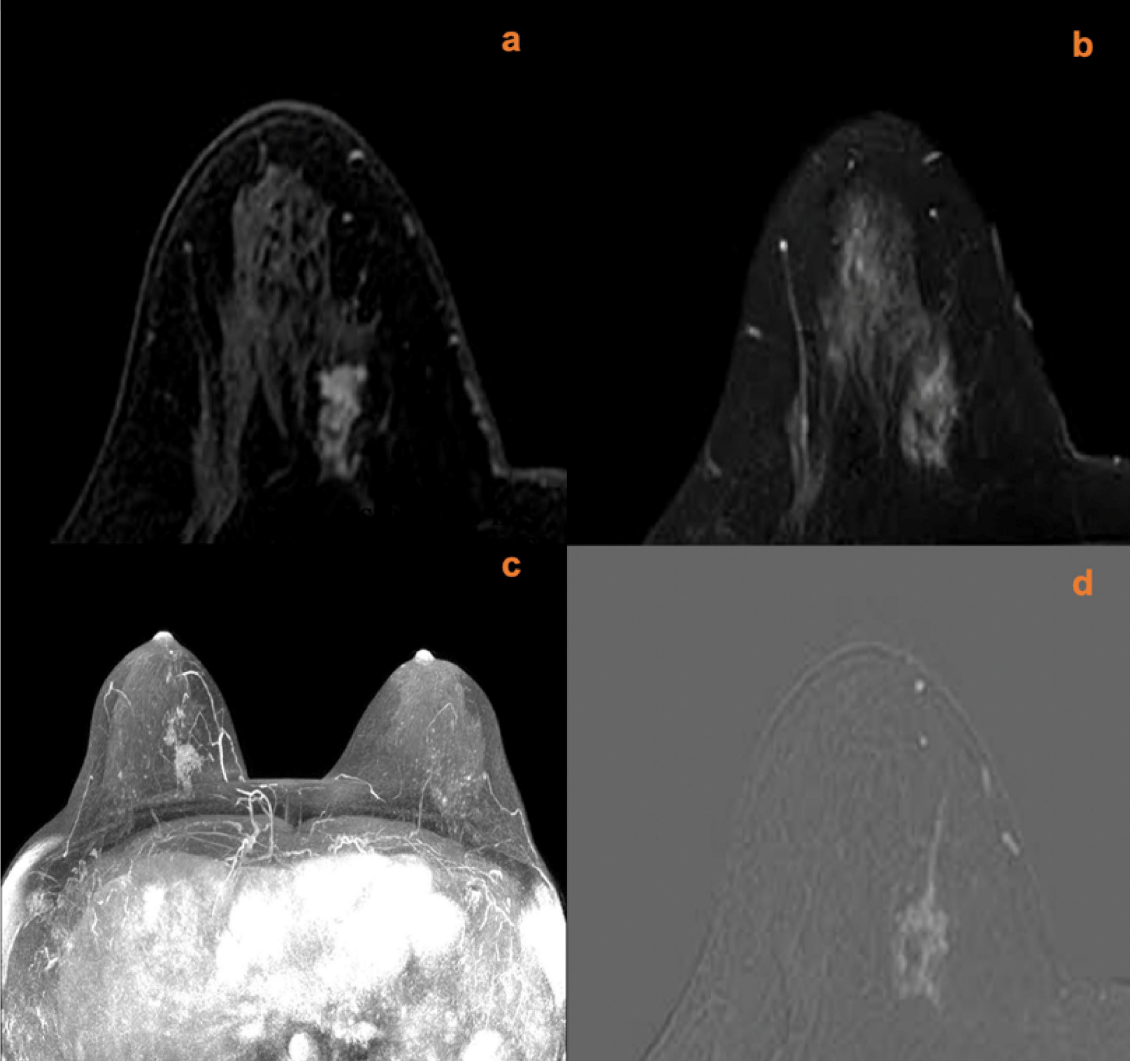
A 40-year-old female with right nipple discharge for two weeks. MRI (**A**, contrast-enhanced axial; **B**, T2w-SPAIR image; **C**, maximum intensity projections image; **D**, subtraction image) shows a right breast non-mass lesion with a root sign. There was no peripheral edema around the lesion. The lesion increased the ipsilateral breast vascularity and the positive AVS sign. The KS of the lesions was 7, while the KS^*^ was 10. Postoperative pathology confirmed that the lesion was an invasive ductal carcinoma.

**Figure 5:**
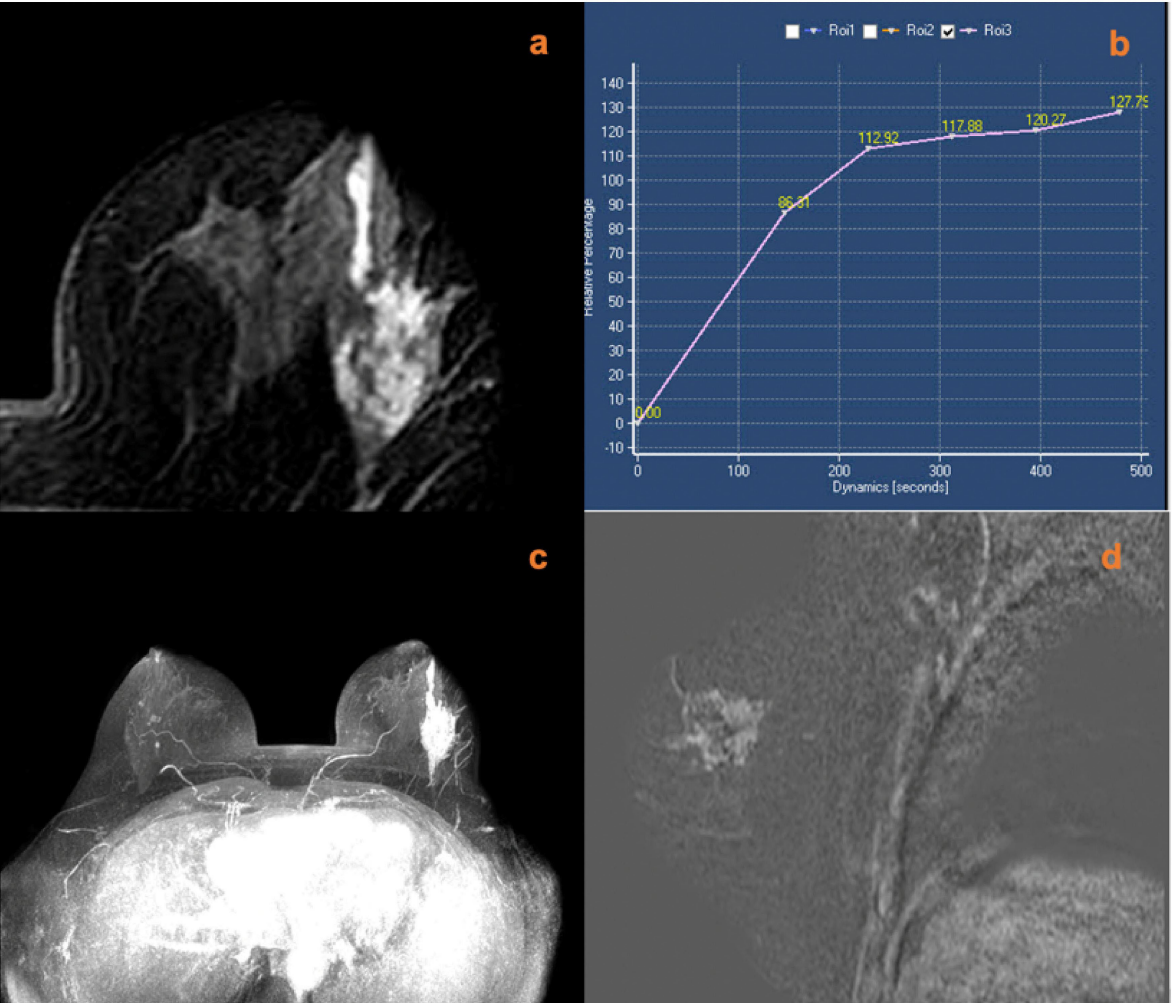
A 45-year-old female was identified with left nipple discharge for one week. MRI (**A**, contrast-enhanced axial; **B**, time signal intensity curve; **C**, maximum intensity projections image; **D**, subtraction image) shows a left breast non-mass lesion with persistent enhancement, increased ipsilateral breast vascularity, and a positive AVS sign. The KS of the lesions was 6, while the KS^*^ was 9. Postoperative pathology confirmed that the lesion was an invasive ductal carcinoma.

A total of 18 lesions had increased scores according to the breast vasculature assessment, including 12 mass lesions and six non-mass lesions. Among these lesions with increased scores, 14 lesions (10 mass lesions and four non-mass lesions) were later confirmed histopathologically as malignant (one ductal carcinoma *in situ*, 12 invasive ductal carcinomas, and one invasive micropapillary carcinoma). The other four lesions were confirmed as benign (two inflammatory lesions, one giant juvenile fibroadenoma, and one benign phylloid tumor). After adjustment for KS, a total of four lesions with an original score below the biopsy threshold had a KS^*^ of >4. Among those lesions, three cases were malignant; all of them were invasive ductal carcinomas. One case was a giant juvenile fibroadenoma. Examples of lesions that had upgraded diagnoses are shown in **Figures 2**–**5**.

### Comparation of the diagnostic performance of KS and KS^*^

**Figure 6:**
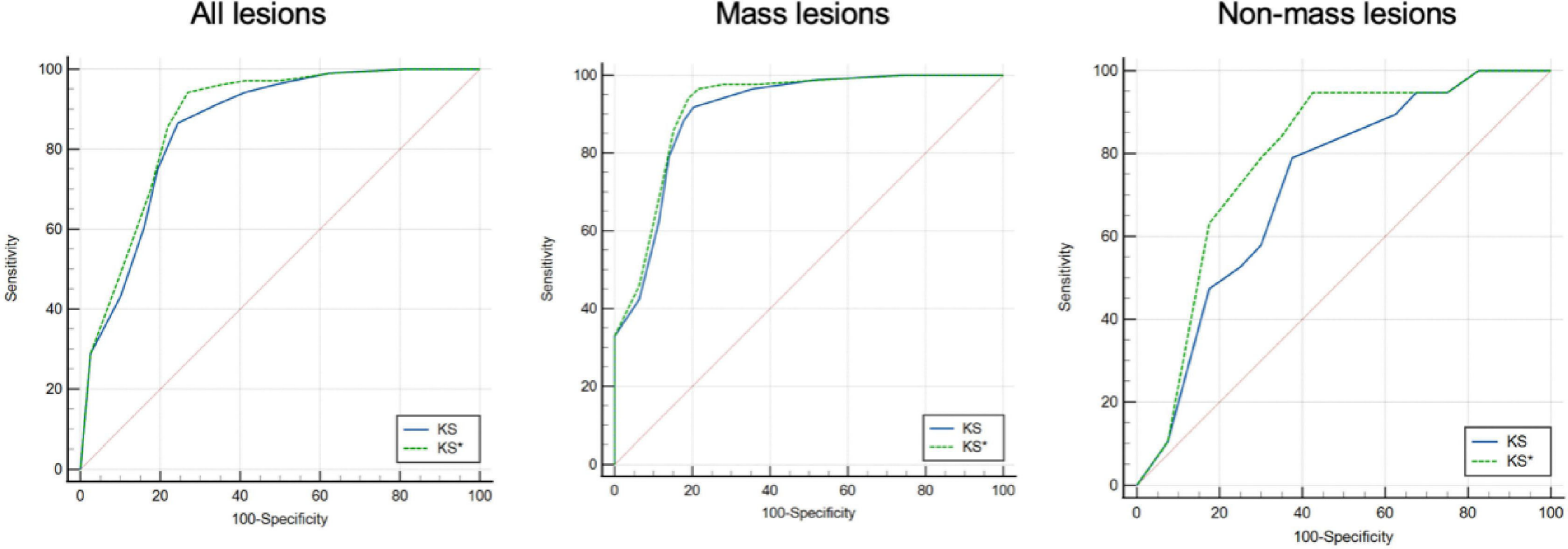
Receiver operating characteristic (ROC) curves for KS* and KS of all lesions [left], mass lesions [middle], and non-mass lesions [right].

For all 223 lesions that were finally included, the AUC of KS^*^ was greater than that of KS (0.877 vs. 0.858, P = 0.016). Subgroup analysis showed that a statistically significant difference in the AUC of KS^*^ and KS was found between mass lesions and non-mass lesions. The largest difference in AUC was observed when analyzing the non-mass subgroup (0.793 vs. 0.725, P = 0.029). Further details of the AUC for the mass and non-mass lesions groups are provided in **Table 5** and **Figure 6**. When a score of >4 was considered a cut-off for malignancy, KS^*^ had a higher sensitivity (97.1%) with a specificity of 58.8%. For KS, the sensitivity was 94.2% and the specificity was 58.8%.

**Table 5:** Comparison of area under the ROC curves of KS and KS^*^ for the mass and non-mass lesions.

	All lesions		Mass lesions		Non-mass lesions	
	AUC	95% CI	AUC	95% CI	AUC	95% CI
KS	0.858	0.805–0.901	0.906	0.851–0.946	0.725	0.593–0.833
KS^*^	0.877	0.827–0.917	0.919	0.867–0.956	0.793	0.668–0.888
	P = 0.016		P = 0.025		P = 0.029	

AUC, the area under the receiver operating characteristics curve.

## Discussion

In this study, we comprehensively investigated the value of breast vascular assessment and KS for the diagnosis of breast cancer, further proposed a combination model based on the above two, defined as KS^*^, and explored its feasibility and effectiveness in differentiating benign from malignant breast lesions. The results revealed that increased ipsilateral breast vascularity and a positive AVS sign were significantly associated with malignancy, and the combination of KS and breast vascular assessment could improve diagnostic performance, especially for non-mass lesions.

KS is a clinical decision tool derived from a complex machine-learning model (15). It is guided by a three-step flowchart based on morphological and dynamically relevant features (presence of spiculations/root signs, enhancement kinetics, type of lesion margin, internal enhancement pattern, and presence of ipsilateral edema) of the lesion (10). The diagnostic score (ranging from 1 to 11) reflects the increasing probability of diagnosing true malignancy. Previous studies showed that KS has a good value in differentiating benign tumors from malignant breast cancers, including various patient groups (9, 10, 12, 14, 15, 27–29). Previous studies confirmed that using KS cannot only achieve a similar or even higher diagnostic effect than ACR-BI-RADS but also reduce unnecessary biopsies (9, 10, 12, 13). Furthermore, KS could improve inter-reader agreement and benefit less experienced radiologists (14). Therefore, it is suggested that KS has a good application prospect in breast MRI.

However, compared with mass lesions, KS is less effective in the diagnosis of non-mass lesions. Woitek et al. evaluated 469 histopathologically verified lesions (270 mass lesions and 199 non-mass lesions) and reported that the AUC for the mass lesions was 0.902 and that for the non-mass lesions was 0.786 (9). Wengert et al. evaluated 167 lesions (51 mass lesions and 116 non-mass lesions) with suspicious mammographic calcifications. The AUC for the mass lesions ranged between 0.904 and 0.963, and the AUC for the non-mass lesions ranged between 0.837 and 0.861 (30). Jajodia et al. evaluated 316 lesions (183 mass lesions and 133 non-mass lesions) for equivocal or inconclusive lesions from mammography and reported that the AUCs for the mass lesions and non-mass lesions were 0.851 and 0.715, respectively (15). Istomin et al. evaluated 697 (555 mass lesions and 142 non-mass lesions). The AUC for the mass lesions ranged between 0.888 and 0.905, and the AUC for the non-mass lesions ranged between 0.742 and 0.749 (12). The results of our study were consistent with many previous studies.

In breast MRI, multifarious benign and malignant lesions can manifest as non-mass enhancement. However, there is a lack of effective methods for the diagnosis of lesions displaying non-mass enhancement (31). The decline in efficiency of KS in the diagnosis of non-mass lesions in our study was consistent with the study by Istomin et al. (12). The root sign is sometimes difficult to evaluate, especially in small mass or non-mass lesions. But the root sign is the most important characteristic in calculating KS. As KS assessment criteria for lesions focus on the morphological and signaling characteristics of the lesion itself, this may be responsible for the reduced diagnostic efficacy of KS for certain breast lesions (e.g., non-mass-enhancing lesions). MRI-based breast vascular assessment can provide information about altered blood supply due to breast lesions, which can be helpful in the diagnosis of malignant lesions.

Angiogenesis and vascular remodeling are considered to be the major regulating events in breast cancer (16). Contrast-enhanced MR angiography can be used to evaluate breast angiogenesis because its contrast enhancement pattern is generated by vascular hyperplasia. Further, including angiography will not prolong the examination time or increase the dose of the contrast agent. According to previous studies, breast cancer was linked with increased ipsilateral breast vascularity and a positive AVS sign (17–19, 32). The elevated vascularity in MR images may have resulted from the tumor’s high metabolism, reduced flow resistance in the tumor blood vessels, angiogenic stimulation in the ipsilateral breast, or a combination of these factors (33). Previously published studies on the diagnostic effect of increased ipsilateral breast vascularity and positive AVS on breast cancer were not consistent. Sibel et al. studied unilateral breast lesions in 102 patients, and the results showed that the sensitivity and specificity of increased ipsilateral breast vascularity were 62% and 79%, respectively. While the sensitivity and specificity of the AVS were 74% and 89%, respectively (33). Verardi et al. studied breast abnormalities in 197 patients and reported that the sensitivity of increased ipsilateral breast vascularity was 74%, the specificity was 94%, and the accuracy was 86% (34). Matthias et al. studied 1,084 histologically verified lesions and reported that the sensitivity of AVS was 47% and the specificity was 94% (19). The results of our study were within the reported ranges. The reported differences in the diagnostic efficacy of increased ipsilateral breast vascularity and positive AVS may have been related to the different study population and different diagnostic criteria used in the evaluation of lesions.

Our study also explored the difference in diagnostic ability due to increased ipsilateral breast vascularity and positive AVS between mass lesions and non-mass lesions, which was rarely elucidated in previous studies. The results showed that no significant difference in the diagnostic efficacy of increased ipsilateral breast vascularity was found between the mass and non-mass lesions. This outcome suggested that increased ipsilateral breast vascularity is less influenced by lesion morphology, which can address the drawbacks of traditional diagnostic methods in the diagnosis of non-mass lesions. The results also showed that the specificity and accuracy of positive AVS in the mass lesions were slightly higher than those in the non-mass lesions. The mass lesions often have more regular boundaries than the non-mass lesions, which might increase the accuracy of using positive AVS.

The KS^*^ was obtained by combining breast vascular assessment and KS to distinguish malignant lesions more accurately from benign lesions. Specifically, the KS scores of those lesions were increased with increased ipsilateral breast vascularity and a positive AVS sign. Most of the lesions with increased scores were confirmed malignant, and three cases of malignant lesions were avoided because of the increased score by extra points. However, a few cases with increased scores were benign lesions, including inflammatory lesions and large solid tumors. This variation may have been related to the false positive by increased blood supply to the breasts. Although malignant lesions are more likely to show a positive AVS sign than benign lesions, solid benign tumors (especially papillomas and phyllodes tumors) and inflammatory changes can also show a positive AVS sign (19). This is because not only malignant tumors but also benign tumors can induce angiogenesis (35). The prevalence of the positive AVS sign in large benign tumors may be related to their higher demand for nutrients and oxygen compared to smaller benign tumors. Similarly, the hyperemia and vasodilation caused by an inflammatory state can be regarded as the histopathological basis of breast angiogenesis caused by inflammatory lesions (36). In our study, the combination of increased ipsilateral breast vascularity and a positive AVS sign had higher specificity than using one of them alone. Therefore, increasing the scores of those lesions by combining the above two parameters may minimize the number of false positives.

According to the instructions, Kaiser scores can be translated into BI-RADS categories as follows: 1–4 translated to BI-RADS 2/3 (≤2% likelihood of malignancy); 5–7 translated to BI-RADS 4 (>2% but <95% likelihood of malignancy); 8–11 translated to BI-RADS 5 (≥95% likelihood of malignancy) (8). The addition of three points for lesions scoring 1-4 and 5-7 enables most lesions corresponding to the BI-RADS classification to be upgraded without causing overdiagnosis. Lesions with an initial Kaiser score greater than or equal to 8 have a higher probability of malignancy. Added scores for such lesions do not affect the diagnosis and are therefore not necessary.

After adjustment with KS, a total of four lesions with an original score below the biopsy threshold had a KS* of >4. Among those lesions, three were malignant. This may account for the elevated sensitivity of KS* over KS. Meanwhile, only a benign lesion with an original score below the biopsy threshold had a KS* of >4 after adjustment with KS. Therefore, the specificity of KS* was not significantly reduced when the threshold was 4.

Combining both methods finally improved the diagnostic value of KS. In our study, KS^*^ had a larger AUC than KS. Although the difference between the ROC analysis results of KS and KS* was not huge in numerical terms, the statistical significance of the difference was present. The largest difference in AUC was observed when assessing the non-mass subgroup. The significantly improved diagnostic value may be related to the relatively low diagnostic efficiency of KS for non-mass lesions. The improvement in diagnostic efficiency for mass lesions is not as obvious as that for non-mass lesions. This difference may be because KS is highly effective in the diagnosis of mass lesions, so many typical mass lesions have appropriate scores before adjustment itself. It is worth noting that the scores of 12 mass lesions were adjusted in our study, and 10 of them were true positives. This outcome suggested that it was still necessary to evaluate breast vascularity in the diagnosis of mass breast lesions using KS.

## Limitations

Our study had several limitations. First, this was a single-center retrospective study, and a possible selection bias might have occurred. However, this study included all consecutive cases that met the inclusion and exclusion criteria within a certain period, which may help reduce selection bias. Second, we did not systematically compare the results of the Kaiser score with those of the ACR-BI-RADS in this study, but the comparison could be found in previous studies. Third, we did not reduce the score of lesions without increased ipsilateral breast vascularity or a positive AVS sign, although this may improve the specificity of the examination. The results of our study and previous studies confirmed that the Kaiser score has a high diagnostic efficiency. For lesions with insufficient evidence of increased blood supply, radical changes in their scores may lead to a misdiagnosis of breast cancer or high-risk lesions. Whether the research results can be promoted and applied still requires further research to confirm.

## Conclusions

The results of this investigation showed that increased ipsilateral breast vascularity and a positive AVS sign were significantly associated with the status of malignancy. The analysis also showed that KS had high efficiency in the differential diagnosis of breast lesions. For lesions that are highly suggestive of suspected malignancy with breast vascular assessment, appropriately increasing their KS scores could reduce missed diagnoses. The combination of breast vascular assessment and KS could be better than KS at diagnosing breast cancer, especially for non-mass lesions.

## Data availability statement

The datasets used and analyzed during the current study are available from the corresponding author on reasonable request.

## Ethics statement

Written informed consent was obtained from the individual(s), and minor(s)’ legal guardian/next of kin, for the publication of any potentially identifiable images or data included in this article.

## Author contributions

X-ZZ: Investigation, data curation, and writing—original draft. L-HL: Formal analysis and validation. SH: Methodology and visualization. H-FY: Supervision. L-PC: Project administration. CD: Resources. S-LL: Resources. X-YZ: Software. HL: Conceptualization, funding acquisition, and writing—review and editing. All authors contributed to the article and approved the submitted version.
